# Production and Biochemical Characterization of Dimeric Recombinant Gremlin-1

**DOI:** 10.3390/ijms23031151

**Published:** 2022-01-21

**Authors:** Stefania Mitola, Cosetta Ravelli, Michela Corsini, Alessandra Gianoncelli, Federico Galvagni, Kurt Ballmer-Hofer, Marco Presta, Elisabetta Grillo

**Affiliations:** 1 Department of Molecular and Translational Medicine, University of Brescia, Viale Europa 11, 25123 Brescia, Italy; cosetta.ravelli@unibs.it (C.R.); michela.corsini@unibs.it (M.C.); alessandra.gianoncelli@unibs.it (A.G.); marco.presta@unibs.it (M.P.); 2 Department of Biotechnology, Chemistry and Pharmacy, University of Siena, 53100 Siena, Italy; federico.galvagni@unisi.it; 3 Biomolecular Research, Paul Scherrer Institute, 5232 Villigen, Switzerland; kurt.ballmer-hofer@unibas.ch

**Keywords:** gremlin-1, dimer, cystine-knot protein, HEK293T expression system

## Abstract

Gremlin-1 is a secreted cystine-knot protein that acts as an antagonist of bone morphogenetic proteins (BMPs), and as a ligand of heparin and the vascular endothelial growth factor receptor 2 (VEGFR2), thus regulating several physiological and pathological processes, including embryonic development, tissue fibrosis and cancer. Gremlin-1 exerts all these biological activities only in its homodimeric form. Here, we propose a multi-step approach for the expression and purification of homodimeric, fully active, histidine-tagged recombinant gremlin-1, using mammalian HEK293T cells. Ion metal affinity chromatography (IMAC) of crude supernatant followed by heparin-affinity chromatography enables obtaining a highly pure recombinant dimeric gremlin-1 protein, exhibiting both BMP antagonist and potent VEGFR2 agonist activities.

## 1. Introduction

The gremlin-1 gene is highly conserved across evolution and, in humans, it encodes for a 184 amino acid long protein, whose theoretical molecular weight is 20.682 Da [1]. Gremlin-1 (hereinafter referred to as gremlin) is a highly basic polypeptide (pI = 9.53), containing 7.61% arginine, 8.7% lysine and 2.17% histidine residues. A signal peptide drives gremlin secretion. Gremlin is found in the endoplasmic reticulum and associated with the cell surface, where it binds to heparan sulfate proteoglycans (HSPGs) once it has been secreted [2]. Gremlin contains three serine residues, which may undergo phosphorylation (Ser77, Ser140 and Ser142). Ser77 was confirmed to be phosphorylated [3]. Putative tyrosine phosphorylation sites are also present. Gremlin is N-glycosylated on Asn42 [2]. However, the biological role of such modifications is not known.

The C-terminal region of gremlin contains nine Cys residues (CX_13_CX_8-9_CX_3_CX_14-18_CX_2_CX_13_CX_15-18_CXC) that show the characteristic spacing of the cystine-knot protein consensus sequence [4]. Crystallographic analyses confirmed that gremlin has the typical folding of cystine-knot proteins, with six Cys residues involved in the formation of the cystine-knot and two Cys residues engaged in an intra-chain disulfide at the fingertips, thus resembling the structure of TGF-β family members [5]. Even though gremlin forms a compact, head-to-tail, non-covalent homodimer in the crystal structure, experimental evidence shows that gremlin forms disulfide-bound homodimers through Cys141 [6].

Gremlin is a pleiotropic protein that belongs to the Cerberus/Dan-related gene family of bone morphogenic protein (BMP) antagonists. By binding and sequestering BMP2/4/7 into inactive complexes, it orchestrates BMP signaling during embryonic development, tissue fibrosis and cancer [7,8,9]. Gremlin is also endowed with BMP-independent activities [10,11,12,13]. Among these, it binds the vascular endothelial growth factor receptor 2 (VEGFR2), eliciting pro-inflammatory and pro-angiogenic responses in tumors and kidney disease [11,12,13,14]. The activation of VEGFR2 on the surface of endothelial cells (ECs) by gremlin triggers EC proliferation, migration and the formation of endothelial sprouts in vitro and in vivo [11]. We have previously shown that the VEGFR2-dependent activity of gremlin is modulated by its oligomeric state. While both states (monomeric and dimeric) bind to VEGFR2, only the dimeric form activates the receptor and angiogenesis. Monomeric gremlin, on the other hand, has been found to act as a VEGFR2 antagonist [6,15]. In addition, the pro-angiogenic activity of gremlin is mediated by its interaction with heparin and HSPGs on cell surfaces, occurring through twelve non-contiguous arginine and lysine residues [16,17]. The high affinity for heparin has previously been exploited for the isolation of gremlin from the culture medium of ECs [3]. Although the BMP antagonist activity of gremlin remains its best characterized feature to date, we now have clear evidence that the biological role of this protein results from a combination of all its distinct activities.

Recombinant gremlin from different sources (e.g., expressed in *E. coli* and refolded, CHO or mouse myeloma cells) has been characterized, providing valuable insight into the structure and mechanism of action of this protein. However, currently available methods for the expression and purification of recombinant gremlin are not best suited to consistently obtain dimeric gremlin that retains full biological activity. Consistent with this observation, a recent study failed to demonstrate that gremlin produced in mouse myeloma cells activates VEGFR2, while being a strong BMP-inhibitor [18]. This is not surprising, as gremlin dimerization is necessary for VEGFR2 activation but not for BMP inhibition [6] and considering that most of the available recombinant gremlin exists in both monomeric and dimeric states, with relatively low dimer vs. monomer ratios which can vary from batch to batch.

In this work, we address, for the first time, the potential role gremlin dimerization may play in obtaining fully active recombinant protein. We set up a lab-scale, multi-step procedure to consistently express and purify micrograms of dimeric gremlin of rat origin (which shares more than 98% amino acid sequence similarity with human gremlin) endowed with full BMP-dependent and VEGFR2-dependent activities. Overall, our protocol provides a reliable framework for attaining native recombinant gremlin that could be exploited for the production of mutated/tagged variants of the protein. Further experiments will be required for scaling up and optimizing the expression/purification procedure to provide higher amounts of fully active protein. Recombinant gremlin could be used in preclinical research studies that aim to further investigate its role as a potential therapeutic target in multiple pathological settings, including obesity, fibrosis and diabetic nephropathy.

## 2. Results

### 2.1. Expression of Recombinant Gremlin in HEK293T Cells

**Figure 1:**
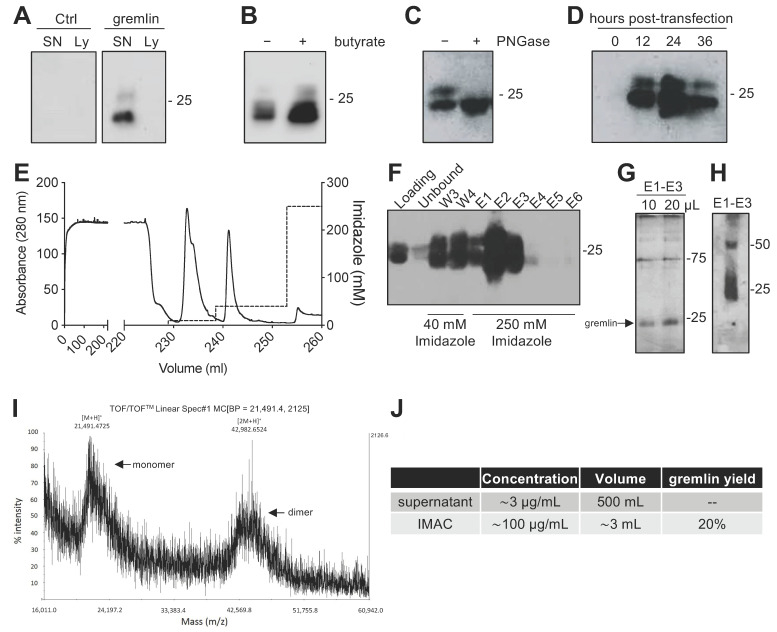
Expression and IMAC purification of recombinant gremlin in HEK293T cells. (**A**) WB analysis of gremlin expression in the supernatant (SN) or the lysate (Ly) of HEK293T cells transiently transfected with pcDNA3-Gremlin-1-HisTag (gremlin) or empty vector (ctrl). (**B**) WB analysis of gremlin expression in the supernatant of HEK293T cells in the absence or the presence of 6 mM sodium butyrate. (**C**) WB analysis of gremlin expressed and secreted by HEK293T cells in the absence or the presence of PNGaseF glycosidase. (**D**) WB analysis of gremlin expressed and secreted by HEK293T cells 0–36 h post transfection. (**E**–**H**) IMAC of recombinant gremlin expressed in the supernatant of HEK293T cells. The chromatogram is shown in (**E**). Fractions were analyzed by WB for gremlin presence (**F**), or by silver staining (**G**). Non-reducing SDS-PAGE followed by anti-gremlin WB of purified gremlin is shown in (**H**). (**I**) MALDI-TOF/TOF–MS spectrum of IMAC-purified recombinant gremlin. Arrows indicate the two species of gremlin that were detected. (**J**) Total yield of IMAC gremlin purification. Data for one representative experiment of at least 3 independent repeats are shown.

Due to the complex structure of gremlin (i.e., intra and inter-molecular disulfides; N-glycosylation), the mammalian HEK293T expression system was chosen to produce the recombinant protein. Adherent HEK293T cells were transiently transfected with a pcDNA3 vector harboring the cDNA of rat gremlin (including the signal peptide-encoding sequence) added with a C-term hexa-histidine tag. As assessed by Western blot (WB), transfected HEK293T cells secreted significant amounts of gremlin (~0.8 μg/mL) into their supernatant. To increase gremlin expression, 6 mM sodium butyrate, a known inhibitor of histone deacetylases and cell proliferation [19,20], was added to the HEK293T cells. Under these circumstances, the yield increased to ~3μg/mL. Gremlin released by HEK293T cells ran in an SDS-PAGE under reducing conditions as two bands of ~24–27 kDa, corresponding to the glycosylated (~30–45%) and non-glycosylated (~70–55%) forms of the protein (Figure 1A,B). Accordingly, a single ~24 kDa band was observed following treatment of recombinant gremlin with the glycosidase PNGaseF (Figure 1C). Following transfection, the peak of protein expression was reached at 24 h post transfection (Figure 1D).

### 2.2. Expression of Recombinant Gremlin in HEK293T Cells Followed by Purification via IMAC Yields Gremlin Both in the Monomeric and Dimeric States

Next, we attempted a medium-scale gremlin production followed by a first-step purification by ion metal affinity chromatography (IMAC). HEK293T cells were transiently transfected in a medium containing 0.5% FCS, and the supernatant (~500 mL) was collected 24 h post transfection, dialyzed against phosphate buffer and loaded onto a Ni-NTA column. As shown in Figure 1E,F, recombinant gremlin eluted from the nickel column at 250 mM imidazole. Although some gremlin was lost in the 40 mM imidazole washing step, purified gremlin was present in the eluted fraction (~24 kDa band) in the absence of significant amounts of contaminant proteins, as assessed by gel silver staining (Figure 1G). Non-reducing SDS-page followed by anti-gremlin WB showed that gremlin eluted both in a monomeric and ~50 kDa disulfide-bound homodimeric forms (Figure 1H). This was further confirmed by MALDI-TOF/TOF–MS (Figure 1I). The main peak, with an *m*/*z* value of approximately 21,500, represented the single charge of the monomer of gremlin, while the *m*/*z* value of about 43,000 represented the single charge of gremlin dimer. The IMAC purification step had an overall yield of 20% (Figure 1J). Similarly, IMAC-purified, 10xhis-tagged gremlin, expressed in murine myeloma cells, existed in both monomeric and dimeric states. The relative amount of dimeric protein differed significantly from batch to batch, and in some cases only reached 1.5% of total gremlin (Figure S1).

### 2.3. IMAC-Purified Gremlin Exhibits Full BMP Antagonist Activity and Only Mild VEGFR2 Activation Capacity

**Figure 2:**
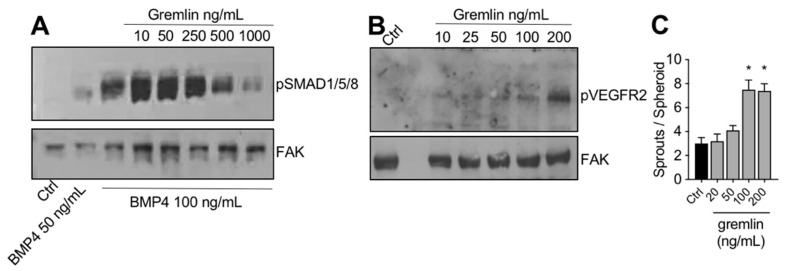
IMAC-purified gremlin retained full BMP-dependent activity and only mild VEGFR2 activation capacity. (**A**) WB analysis of phospho-SMAD1/5/8 levels in HepG2 cells stimulated with BMP4 in the presence or the absence of indicated doses of IMAC-purified gremlin. FAK, loading control. (**B**) WB analysis of phospho-VEGFR2 levels in HUVE cells stimulated with increasing doses of IMAC-purified gremlin. FAK, loading control. (**C**) HUVE cells sprouting assay in the absence or the presence of increasing doses of IMAC-purified gremlin. Data are expressed as mean ± SEM. *, *p* < 0.05 Student’s *t* test vs. untreated cells. Data from one representative experiment of at least 3 independent repeats are shown.

The IMAC-purified gremlin was tested for its biological activities. In a first set of experiments, to verify its BMP antagonist activity, hepatocarcinoma HepG2 cells were stimulated with BMP4 in the absence or the presence of increasing doses of IMAC-purified gremlin and assessed for phospho-SMAD1/5/8 levels by WB. As expected, IMAC-purified gremlin induced a dose-dependent inhibition of BMP4-driven SMAD phosphorylation (Figure 2A). We obtained similar results with all different IMAC gremlin preparations (data not shown). On the contrary, not all batches of IMAC gremlin induced a significant activation of VEGFR2 or of the VEGFR2-dependent biological activities in the human umbilical vein endothelial cells (HUVEC) (data not shown). Figure 2B,C shows the weak activation of VEGFR2 obtained with one of the different batches of purified IMAC gremlin that were tested. Indeed, VEGFR2 and angiogenesis were only activated when high doses of IMAC-purified gremlin (100–200 ng/mL) were used.

### 2.4. Purification of Fully Active, Dimeric Gremlin by Heparin-Affinity Chromatography

**Figure 3:**
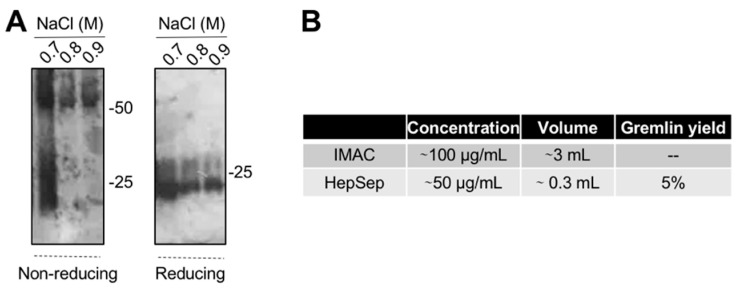
Heparin-affinity chromatography allows isolating pure dimeric recombinant gremlin. IMAC-purified gremlin was subjected to a second step of purification through heparin-affinity chromatography. (**A**) WB analysis under non-reducing and reducing conditions of fractions eluted from the heparin-affinity chromatography. (**B**) Total yield of gremlin purification through heparin-affinity chromatography. Data from one representative experiment of at least 3 independent repeats are shown.

The varying and low ability of the IMAC-purified gremlin to activate VEGFR2 suggests the possibility that some of the protein may be present in an unfolded, biologically inactive form. This prompted us to introduce an additional purification step. We exploited heparin-affinity chromatography, as the heparin binding domain of gremlin consists of a non-linear 12-aa sequence [17] that allowed us to isolate the correctly folded and fully active gremlin protein. IMAC-purified gremlin was loaded on a heparin-sepharose (HepSep) column and eluted with a 0.2 to 1.2 M NaCl step gradient. WB analysis demonstrated that the 0.7 M NaCl fraction contained gremlin monomers together with dimers probably not correctly folded (Figure 3A), whereas gremlin eluting at higher ionic strength (0.8–0.9 M NaCl) ran on a non-reducing SDS-PAGE as a ~50 kDa dimer. The net yield of this second purification step was ~5% (Figure 3B).

**Figure 4:**
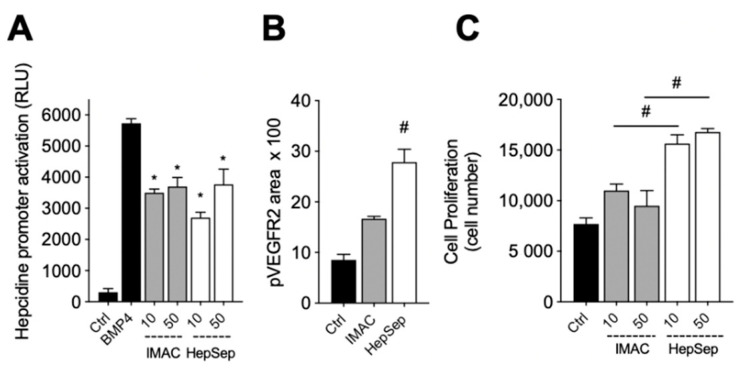
Pure dimeric gremlin exhibits strong biological activity. (**A**) Activation of Hepcidin promoter in a luciferase reporter assay upon BMP4 stimulation (50 ng/mL). When indicated, BMPs were pre-incubated in the absence, or presence, of the indicated concentration (ng/mL) of IMAC-purified or dimeric heparin-sepharose (HepSep)-purified gremlin. (**B**) pVEGFR2 accumulation into the VPM of GM7373-VEGFR2 endothelial cells adherent to IMAC-purified or dimeric HepSep-purified gremlin (dishes were coated with 2 µg/mL of recombinant proteins). (**C**) Cell proliferation of HUVE cells stimulated with increasing doses (ng/mL) of IMAC- or HepSep-purified gremlin. Data are expressed as mean ± SEM of three independent experiments. *, *p* < 0.05 Student’s *t* test vs. BMP-treated cells. #, *p* < 0.05 Student’s *t* test vs. gremlin IMAC-treated cells.

We next measured the bioactivity of HepSep-purified dimeric gremlin. As shown in Figure 4A, HepSep gremlin blocks the BMP4-dependent activation of the hepcidin promoter in a luciferase reporter assay, at an extent similar to that of IMAC-purified gremlin. On the contrary, dimeric gremlin induced a significantly more potent response than IMAC-purified gremlin in terms of VEGFR2 activation, measured as phosphorylated receptor accumulated on the ventral plasmatic membrane (VPM), and in terms of proliferation of HUVECs (Figure 4B,C). These results are consistent with our previous findings showing that monomeric and dimeric gremlin antagonize BMPs in a similar manner, while only dimeric gremlin induces the full activation of ECs via VEGFR2 [6].

## 3. Discussion

Gremlin is a pleiotropic protein that plays a pivotal role in several human diseases. Recent studies have addressed its pathological role in adipose tissue, kidney disease and cancer [8,21,22]. Such studies benefited from the availability of a recombinant form of gremlin that has contributed to rapid advancements in gremlin-related research. Recombinant gremlin has been characterized from different sources (e.g., expressed in *E. coli* and refolded, expressed in CHO or mouse myeloma cells). However, we, and others, observed that gremlin displays high batch-to-batch variability and sometimes completely lacks VEGFR2-dependent activity [15,18]. This may relate to the complex structure of gremlin, which requires both intramolecular and intermolecular disulfide bridges and other post-translational modifications (i.e., N-glycosylation). Thus, we used to test each batch prior to performing experiments.

In the present study, we report a novel approach for the production of recombinant gremlin and address, for the first time, the importance of the dimeric state of the protein for obtaining a fully bioactive recombinant gremlin. The results show that the biological properties of recombinant gremlin relate to differences in the relative amount of dimeric vs. monomeric forms present in different batches. Indeed, only dimeric gremlin exhibits VEGFR2-dependent activity, whereas gremlin dimerization is not required for BMP inhibition [6]. Additionally, monomeric gremlin acts as a VEGFR2 antagonist [6,15]. On this basis, we speculate that the results obtained in a recent study showing that gremlin retained its BMP inhibitory activity, whilst being devoid of VEGFR2 agonist activity [18], were due to the fact that the recombinant protein was mostly in a monomeric form.

Here, the mammalian HEK293T expression system was exploited for the production of recombinant gremlin to favor its correct post-translational modification (e.g., glycosylation). HEK293T cells yield significant amounts of gremlin secreted into the supernatant. Secreted gremlin was post translationally modified (i.e., glycosylation), possibly leading to increased biochemical stability. Furthermore, gremlin was present both in a monomeric and dimeric state, suggesting that, when expressed in HEK293T cells, it displays, at least in part, the native disulfide arrangement. Next, we set up a two-step purification procedure. Our results demonstrate that gremlin purified via IMAC is active as a BMP antagonist and retains a none-to-mild capacity to activate VEGFR2. This finding may be due to the relatively low dimer vs. monomer ratio (approximately 20%). Gremlin purified via heparin-affinity chromatography is, however, highly enriched in the dimeric form and displays a strong activity on VEGFR2. It is important to note that the heparin binding site of gremlin consists of a conformational arrangement of lysine and arginine residues [17]. Thus, heparin-affinity chromatography is essential to isolate the correctly folded native recombinant gremlin. Consistent with this data, pro-angiogenic gremlin was originally isolated from the conditioned medium of ECs via heparin-affinity chromatography [3].

Our protocol provides a fast and scalable procedure for obtaining bioactive dimeric gremlin. The procedure requires simple equipment that is available in most research laboratories. Attempts to increase the yield of gremlin expression in HEK293T cells should consider suspension cultures and bio-reactors. One additional approach may be the co-expression of gremlin with chaperones and/or disulfide isomerases [23,24] to facilitate gremlin folding and dimerization.

## 4. Materials and Methods

### 4.1. Cell Cultures

Human embryonic kidney HEK293T/17 (ATCC) cells, human hepatocellular carcinoma HepG2 (ATCC) cells and fetal bovine aortic endothelial GM7373 cells overexpressing VEGFR2 [25] were grown in Dulbecco modified Eagle’s medium (DMEM, Gibco Life Technologies, Grand Island, NY, USA) containing 10% fetal calf serum (FCS, Gibco Life Technologies). Human umbilical vein endothelial cells (HUVECs) were grown in M199 medium (Gibco Life Technologies), supplemented with 20% FCS, endothelial cell growth factors (100 µg/mL, Sigma-Aldrich, St. Louis, MO, USA) and porcine heparin (100 µg/mL, Sigma-Aldrich).

### 4.2. Generation of DNA Constructs

The cDNA sequence of rat gremlin-1 (including the signal peptide-encoding sequence) was amplified from pMEX-Gremlin-1 (NM_019282.3) (provided by L. Topol [26]) and cloned into pcDNA3 vector (ThermoFisher Scientific, Waltham, MA, USA) upstream to a 6xHis-Tag sequence to obtain pcDNA3-Gremlin-1-HisTag. Of note, rat gremlin-1 shares 98.4% protein sequence similarity with human gremlin-1, only differing for 2/159 amino acid residues in the mature protein.

### 4.3. Expression in HEK293T Cells and Purification of Recombinant Gremlin

pcDNA3-Gremlin-1-HisTag was used to transiently transfect adherent HEK293T using polyethyleneimine (PEI, Polysciences, Inc., Warrington, PA, USA). Transfection was carried out in DMEM supplemented with 0.5% FCS. When indicated, 6 mM sodium butyrate was added to the cell culture medium. After transfection (0–36 h), the medium was collected, clarified and dialyzed against a 20 mM phosphate buffer (pH 8.0) containing 0.5 M NaCl (binding buffer). Gremlin was purified from the dialyzed medium by IMAC on a 1.0 mL HiTrap Chelating HP Ni^2+^-column (Cytiva, Marlborough, MA, USA), pre-equilibrated with binding buffer, using AKTA™ start FPLC system (Cytiva). The column was eluted with a discontinuous imidazole gradient (10, 40 and 250 mM). Gremlin eluted at 250 mM imidazole. Finally, IMAC-purified gremlin was buffer exchanged with PBS for biological activity assays. When indicated, IMAC-purified gremlin was loaded onto a 50–100 μL heparin-sepharose column, packed into a glass Pasteur pipette and eluted with a discontinuous NaCl gradient (0.3–1.2 M NaCl). Fractions of interest were collected and buffer exchanged with PBS. Proteins were quantified by measuring the OD at 280 nm.

For preparative gremlin purification, 20 culture dishes (∅15 cm) of 70% confluent HEK293T cells were transfected as described above in 25 mL of medium/dish. Twenty-four hours post transfection, 500 mL of supernatant was collected and dialyzed against 5.0 L of phosphate buffer and purified by IMAC. IMAC gremlin was further purified by heparin-affinity chromatography. The heparin column was washed with PBS containing 0.6 M NaCl and then eluted with PBS containing 1.2 M NaCl. Finally, purified gremlin was buffer exchanged with PBS. When indicated, gremlin was incubated with 10 u of PNGaseF glycosidase for 18 h at 4 °C and further analyzed by Western blot (WB).

### 4.4. Gel Analyses and Western Blotting

Cell lysates, supernatants or purified proteins (including recombinant human gremlin-1 purchased from R&D) were separated by SDS-PAGE under reducing (+dithiothreitol, DTT) or non-reducing (-DTT) conditions. Gels were either stained with silver staining or further processed for WB. To this, gels were transferred to PVDF membranes and incubated with the indicated primary antibodies followed by incubation with specific HRP-conjugated secondary antibodies. A chemiluminescent signal was acquired using the ChemiDoc Imaging System (BioRad, Hercules, CA, USA).

### 4.5. Matrix-Assisted Laser Desorption/Ionization (MALDI) Time-Of-Flight (TOF)/TOF Mass Spectrometry (MS) (MALDI-TOF/TOF–MS) Analyses

IMAC-purified gremlin was dissolved in 0.1% TFA and mixed with sinapic acid (SA) (10 mg/mL) in acetonitrile:water:TFA (70.0:30.0:0.1, *v*/*v*/*v*). An amount of 1 μL of this preparation was applied to the MALDI plate and allowed to dry at room temperature. Experiments were performed using an AB Sciex 5800 MALDI-TOF/TOF–MS, equipped with a nitrogen laser (k = 337 nm). Samples were measured in linear mode, setting the laser intensity at 5000 μJ with a pulse laser of 400 Hz, the detector voltage multiplier at 0.68, and recording the spectra in a mass range from 10,000 to 70,000 Da with a focus mass of 22,000 Da, providing information on the presence of monomer and dimer. A ProteoMassTM Protein MALDI-MS Calibration Kit was used for calibration.

### 4.6. Analysis of SMAD and VEGFR2 Activation

HepG2 cells were made quiescent by a 3 h starvation in DMEM without FCS and stimulated for 15 min with indicated doses of BMP4 pre-incubated in the absence or the presence of the indicated doses of recombinant gremlin. Alternatively, HUVECs were made quiescent by a 20 h starvation and stimulated with the indicated doses of gremlin for 10 min. After the treatment, cells were lysed in 50 mM Tris–HCl buffer (pH 7.4) containing 1.0% Triton-X 100, 0.1% BriJ, 1.0 mM sodium orthovanadate and protease inhibitor cocktail. An amount of 100 µg of proteins was subjected to SDS-PAGE followed by WB by using anti-phospho-SMAD1/5/8 (Cell Signaling, Danvers, MA, USA), anti-phospho-VEGFR2 (Y1175) (ThermoFisher Scientific, Waltham, MA, USA) or anti-FAK (Santa Cruz, Dallas, TX, USA) antibodies.

### 4.7. Luciferase Reporter Gene Assay

HepG2 cells were transiently transfected with pGL2-Luc-HAMP vector harboring BMP-responsive hepcidin promoter upstream to a Firefly luciferase reporter gene (kindly provided by Prof. Maura Poli, DMMT, University of Brescia, Brescia, Italy). Twelve hours after transfection, serum-starved cells were stimulated with 50 ng/mL of BMP4 (R&DSystem, Minneapolis, MN, USA) for 16 h in the absence or in the presence of increasing concentrations of recombinant gremlin. Cells were then lysed, and luciferase activity was determined with the ONE-Glo™ Luciferase Assay System (Promega, Madison, WI, USA). Firefly luciferase activity was measured with an EnSight Multi-mode Plate Reader (PerkinElmer, Waltham, MA, USA).

### 4.8. Ventral Plasma Membrane Preparation

Coverslips were coated with recombinant gremlin at indicated doses, and GM7373-VEGFR2 cells were allowed to adhere for 1 h. To prepare ventral plasma membranes, (VPMs) cells were washed and squirted over by a jet of ice-cold water. The remaining VPMs were fixed and analyzed for VEGFR2 activation with anti-pVEGFR2 antibody by immunofluorescence [25]. VPMs were photographed using an Axiovert 200 M epifluorescence microscope equipped with a Plan-Apochromat 63X/1.4 NA oil objective (Zeiss, Oberkochen, Germany). Images were analyzed by FiJi software (http://rsbweb.nih.gov/ij, accessed on 15 November 2021).

### 4.9. HUVEC Proliferation

Sub-confluent HUVECs were made quiescent by a 20 h starvation and incubated for 48 h with the indicated doses of gremlin. At the end of the incubation, cells were trypsinized and counted.

### 4.10. Endothelial Cell Sprouting Assay

HUVEC spheroids were prepared in 20% methylcellulose medium, embedded in fibrin gel, and stimulated with indicated doses of gremlin. The formation of radially growing cell sprouts was observed and counted after 24 h [27].

## 5. Conclusions

To overcome the lack of full activity and batch-to-batch variability of recombinant gremlin, we set up a reproducible, lab-scale and multi-step procedure for the expression and purification of bioactive gremlin in mammalian cells. Remarkably, we address, for the first time, the importance of gremlin dimerization for obtaining fully active gremlin. Further efforts are warranted to scale up the production of dimeric protein.

## Data Availability

Not applicable.

## Supplementary Materials

The following supporting information can be downloaded at: https://www.mdpi.com/article/10.3390/ijms23031151/s1.

## References

[1] Topol L., Modi W., Koochekpour S., Blair D. (2000). DRM/GREMLIN (CKTSF1B1) maps to human chromosome 15 and is highly expressed in adult and fetal brain. Cytogenet. Cell Genet..

[2] Topol L.Z., Bardot B., Zhang Q., Resau J., Huillard E., Marx M., Calothy G., Blair D.G. (2000). Biosynthesis, Post-translation Modification, and Functional Characterization of Drm/Gremlin. J. Biol. Chem..

[3] Stabile H., Mitola S.M.F., Moroni E., Belleri M., Nicoli S., Coltrini D., Peri F., Pessi A., Orsatti L., Talamo F. (2007). Bone morphogenic protein antagonist Drm/gremlin is a novel proangiogenic factor. Blood.

[4] Avsian-Kretchmer O., Hsueh A.J.W. (2004). Comparative Genomic Analysis of the Eight-Membered Ring Cystine Knot-Containing Bone Morphogenetic Protein Antagonists. Mol. Endocrinol..

[5] Kisonaite M., Wang X., Hyvönen M. (2016). Structure of Gremlin-1 and analysis of its interaction with BMP-2. Biochem. J..

[6] Grillo E., Ravelli C., Corsini M., Ballmer-Hofer K., Zammataro L., Oreste P., Zoppetti G., Tobia C., Ronca R., Presta M. (2016). Monomeric gremlin is a novel vascular endothelial growth factor receptor-2 antagonist. Oncotarget.

[7] Michos O., Panman L., Vintersten K., Beier K., Zeller R., Zuniga A. (2004). Gremlin-mediated BMP antagonism induces the epithelial-mesenchymal feedback signaling controlling metanephric kidney and limb organogenesis. Development.

[8] Ren J., Smid M., Iaria J., Salvatori D.C.F., van Dam H., Zhu H.J., Martens J.W.M., Dijke P.T. (2019). Cancer-associated fibroblast-derived Gremlin 1 promotes breast cancer progression. Breast Cancer Res..

[9] Koli K., Myllãrniemi L.M., Vuorinen K., Salmenkivi K., Ryynänen M.J., Kinnula V.L., Keski-Oja J. (2006). Bone Morphogenetic Protein-4 Inhibitor Gremlin Is Overexpressed in Idiopathic Pulmonary Fibrosis. Am. J. Pathol..

[10] Chen B., Blair D.G., Plisov S., Vasiliev G., Perantoni A.O., Chen Q., Athanasiou M., Wu J.Y., Oppenheim J.J., Yang D. (2004). Cutting Edge: Bone Morphogenetic Protein Antagonists Drm/Gremlin and Dan Interact with Slits and Act as Negative Regulators of Monocyte Chemotaxis. J. Immunol..

[11] Mitola S.M.F., Ravelli C., Moroni E., Salvi V., Leali D., Ballmer-Hofer K., Zammataro L., Presta M. (2010). Gremlin is a novel agonist of the major proangiogenic receptor VEGFR2. Blood.

[12] Corsini M., Moroni E., Ravelli C., Andrés G., Grillo E., Ali I.H., Brazil D.P., Presta M., Mitola S. (2014). Cyclic Adenosine Monophosphate-Response Element–Binding Protein Mediates the Proangiogenic or Proinflammatory Activity of Gremlin. Arter. Thromb. Vasc. Biol..

[13] Lavoz C., Alique M., Díez R.R., Pato J., Keri G., Mezzano S., Egido J., Ruiz-Ortega M. (2015). Gremlin regulates renal inflammation via the vascular endothelial growth factor receptor 2 pathway. J. Pathol..

[14] Mitola S., Moroni E., Ravelli C., Andres G., Belleri M., Presta M. (2008). Angiopoietin-1 mediates the proangiogenic activity of the bone morphogenic protein antagonist Drm. Blood.

[15] Rowan S.C., Piouceau L., Cornwell J., Li L., McLoughlin P. (2018). EXPRESS: Gremlin1 blocks vascular endothelial growth factor signalling in the pulmonary microvascular endothelium. Pulm. Circ..

[16] Chiodelli P., Mitola S., Ravelli C., Oreste P., Rusnati M., Presta M. (2011). Heparan Sulfate Proteoglycans Mediate the Angiogenic Activity of the Vascular Endothelial Growth Factor Receptor-2 Agonist Gremlin. Arter. Thromb. Vasc. Biol..

[17] Tatsinkam A.J., Mulloy B., Rider C. (2015). Mapping the heparin-binding site of the BMP antagonist gremlin by site-directed mutagenesis based on predictive modelling. Biochem. J..

[18] Dutton L.R., O’Neill C.L., Medina R.J., Brazil D.P. (2019). No evidence of Gremlin1-mediated activation of VEGFR2 signaling in endothelial cells. J. Biol. Chem..

[19] Liu Y., Zhou X., Song Z., Zhang Y. (2014). Sodium butyrate enhances the acidic isoform content of recombinant human erythropoietin produced by Chinese hamster ovary cells. Biotechnol. Lett..

[20] Grünberg J., Knogler K., Waibel R., Novak-Hofer I. (2003). High-Yield Production of Recombinant Antibody Fragments in HEK-293 Cells Using Sodium Butyrate. Biotechniques.

[21] Hedjazifar S., Shahidi R.K., Hammarstedt A., Bonnet L., Church C., Boucher J., Blüher M., Smith U. (2020). The Novel Adipokine Gremlin 1 Antagonizes Insulin Action and Is Increased in Type 2 Diabetes and NAFLD/NASH. Diabetes.

[22] Yang Y., Zeng Q.-S., Zou M., Zeng J., Nie J., Chen D., Gan H.-T. (2021). Targeting Gremlin 1 Prevents Intestinal Fibrosis Progression by Inhibiting the Fatty Acid Oxidation of Fibroblast Cells. Front. Pharmacol..

[23] Cain K., Peters S., Hailu H., Sweeney B., Stephens P., Heads J., Sarkar K., Ventom A., Page C., Dickson A. (2013). A CHO cell line engineered to express XBP1 and ERO1-Lalpha has increased levels of transient protein expression. Biotechnol. Prog..

[24] Borth N., Mattanovich D., Kunert R., Katinger H. (2005). Effect of Increased Expression of Protein Disulfide Isomerase and Heavy Chain Binding Protein on Antibody Secretion in a Recombinant CHO Cell Line. Biotechnol. Prog..

[25] Ravelli C., Grillo E., Corsini M., Coltrini D., Presta M., Mitola S. (2015). β3 Integrin Promotes Long-Lasting Activation and Polarization of Vascular Endothelial Growth Factor Receptor 2 by Immobilized Ligand. Arter. Thromb. Vasc. Biol..

[26] Topol L.Z., Marx M., Laugier D., Bogdanova N.N., Boubnov N.V., A Clausen P., Calothy G., Blair D.G. (1997). Identification of drm, a novel gene whose expression is suppressed in transformed cells and which can inhibit growth of normal but not transformed cells in culture. Mol. Cell. Biol..

[27] Di Somma M., Schaafsma W., Grillo E., Vliora M., Dakou E., Corsini M., Ravelli C., Ronca R., Sakellariou P., Vanparijs J. (2019). Natural Histogel-Based Bio-Scaffolds for Sustaining Angiogenesis in Beige Adipose Tissue. Cells.

