# Supplementary material for: Production and Biochemical Characterization of Dimeric Recombinant Gremlin-1
Source: Int J Mol Sci. 2022 Jan 21;23(3):1151. doi: 10.3390/ijms23031151 (PMC8835488; doi:10.3390/ijms23031151)
Supplement: Supplementary file 1 [file ijms-23-01151-s001.zip › ijms-1555235-supplementary.pdf]

Production and biochemical characterization of dimeric recombinant Gremlin-1

Stefania Mitola[1]*, Cosetta Ravelli[1], Michela Corsini[1], Alessandra Gianoncelli[1], Federico Galvagni[2], Kurt Ballmer-Hofer[3], Marco Presta[1] and Elisabetta Grillo[1]*

[1] Department of Molecular and Translational Medicine, University of Brescia, Brescia, 25123, Italy
[2] Department of Biotechnology, Chemistry and Pharmacy, University of Siena, 53100 Siena, Italy
[3] Biomolecular Research; Molecular Cell Biology; Paul Scherrer Institut; Villigen, 5232, Switzerland

Figure S1

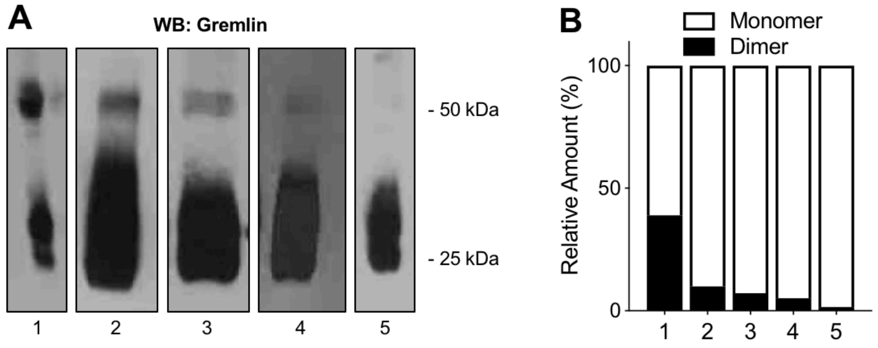

Analysis of the oligomeric state of recombinant gremlin. A, WB analysis of gremlin oligomeric state by non-reducing SDS-PAGE in 5 distinct batches of recombinant human gremlin produced in mouse myeloma cells. **B**, WB densitometry.
